# Knockdown of Golgi phosphoprotein 73 blocks the trafficking of matrix metalloproteinase‐2 in hepatocellular carcinoma cells and inhibits cell invasion

**DOI:** 10.1111/jcmm.14055

**Published:** 2019-01-24

**Authors:** Yiming Liu, Xiaodi Zhang, Sining Zhou, Jieyao Shi, Yun Xu, Jia He, Feng Lin, Anbang Wei, Linfu Zhou, Zhi Chen

**Affiliations:** ^1^ State Key Laboratory for Diagnosis and Treatment of Infectious Diseases Collaborative Innovation Center for Diagnosis and Treatment of Infectious Disease The First Affiliated Hospital Zhejiang University School of Medicine Hangzhou China; ^2^ Department of Biochemistry and Molecular Biology Zhejiang University School of Medicine Hangzhou China; ^3^ Department of Pathology and Pathophysiology Program in Molecular Cell Biology Zhejiang University School of Medicine Hangzhou China; ^4^ Institute of Immunology Zhejiang University School of Medicine Hangzhou China; ^5^ Key Laboratory of Clinical In Vitro Diagnostic Techniques of Zhejiang Province Department of Clinical Laboratory the First Affiliated Hospital Zhejiang University School of Medicine Hangzhou China

**Keywords:** GP73, hepatocellular carcinoma, intrahepatic metastasis, MMP‐2, negative feedback loop, protein trafficking

## Abstract

Golgi phosphoprotein 73 (GP73) has been regarded as a novel serum biomarker for the diagnosis of hepatocellular carcinoma (HCC) in recent years. It has been reported that the upregulation of GP73 may promote the carcinogenesis and metastasis of HCC; however, the mechanisms remain poorly understood. In this study, GP73 correlates positively with matrix metalloproteinase‐2 (MMP‐2) in HCC‐related cells and tissues. Further studies indicate that the knockdown of GP73 blocks MMP‐2 trafficking and secretion, resulting in cell invasion inhibition. Additionally, the knockdown of GP73 induces the accumulation of intracellular MMP‐2, which inhibits the phosphorylation of Src at Y416 and triggers the inhibition of SAPK/JNK and p53‐p21 signalling pathways through a negative feedback loop. Finally, the transactivation of *MMP2* was inhibited by the reduction in E2F1. This study reveals that GP73 plays functional roles in the trafficking and equilibrium of epithelial‐mesenchymal transition (EMT)‐related secretory proteins and that GP73 serves as a new potential target for combating the metastasis of HCC.

## 1 INTRODUCTION

Hepatocellular carcinoma (HCC) is the third leading cause of cancer‐related death worldwide, with limited treatment options.^1^, ^2^, ^3^ HBV or HCV infections increase the occurrence of HCC, and statistically, more than 80% of HCC patients are HBV or HCV infected.^2^, ^3^ Recurrence and metastasis are the main causes of HCC‐related deaths.^4^ HCC cells metastasize rapidly through the vasculature, and intrahepatic metastasis is the common form of metastasis.^5^ Robust estimates suggest that approximately 70% of HCC patients will relapse within 5 years following surgery.^6^ Therefore, it is critical to diagnose and control HCC metastasis as soon as possible to extend the survival time of HCC patients.

GP73 is a highly phosphorylated protein located in the Golgi apparatus that can also be cleaved and secreted into serum.^7^, ^8^ GP73 is highly expressed in several kinds of cancers, including HCC, nonsmall cell lung cancer, breast carcinoma, pancreatic carcinoma, melanoma, and prostate carcinoma.^9^, ^10^, ^11^, ^12^, ^13^ As the expression of GP73 was positively associated with the process of cancers, this protein has been regarded as a novel serum biomarker in the diagnosis of cancers, especially in HCC diagnosis.^9^, ^14^, ^15^

Recently, it was also reported that serum GP73 facilitates the progression of HBV‐related acute and chronic hepatitis, acute‐on‐chronic liver failure, fibrosis, and other immunologically mediated liver diseases.^16^, ^17^, ^18^, ^19^, ^20^ Furthermore, some studies even indicate that GP73 is a suitable diagnostic biomarker not only for HCC but also for liver cirrhosis.^21^, ^22^

Although studies indicate that GP73 is a remarkable biomarker in clinical diagnostics, its function remains unclear. In recent years, some studies have indicated that highly expressed GP73 promotes the migration and invasion of HCC,^8^, ^23^ but the molecular processes are far more complex.

MMP‐2 is a critical member of the matrix metalloproteinase family, which is activated on the plasma membrane and secreted into the extracellular space, resulting in fibrillin degradation and extracellular matrix damage.^24^, ^25^, ^26^ MMP‐2 is often highly expressed in cancer cells, facilitating cell invasion. The transcription, activation, secretion, and function of MMP‐2 have been studied in recent years, but the process of intracellular MMP‐2 trafficking is still poorly understood.^24^

In this study, the knockdown of GP73 blocked intracellular MMP‐2 trafficking, resulting in cell invasion inhibition. Moreover, the knockdown of GP73 triggered SAPK/JNK and p53‐p21 signalling pathways inhibition, which modulated the equilibrium of intracellular MMP‐2 by regulating the transactivation of *MMP2*. These studies provided a potential target for combating metastatic HCC.

## 2 MATERIALS AND METHODS

### 2.1 Reagents

Dulbecco's modified Eagle's medium (DMEM), foetal bovine serum (FBS), Lipofectamine 3000, Lipofectamine RNAi Max, and puromycin were purchased from Thermo Fisher (Carlsbad, CA, USA). The restriction enzymes BamHI, EcoRI, HindIII, KpnI, and SacI were purchased from TaKaRa (Shiga, Japan). BFA, Saracatinib, and PP1 were obtained from MedChem Express Co., Ltd. (Shanghai, China).

### 2.2 Antibodies

Rabbit polyclonal antibodies against GP73 were obtained from Thermo Fisher, and mouse monoclonal antibodies against GP73 were purchased from Abnova (Taipei, Taiwan). Antibodies against MMP‐2, CD81, and CD63 were obtained from Abcam (Cambridge, MA, USA). Antibodies against β‐actin, flag, and HA were purchased from EarthOx (San Francisco, CA, USA), and antibodies against LaminB were purchased from Proteintech Inc (Wuhan, China). Antibodies against Src, p‐Src(Y416), SAPK/JNK, p‐SAPK/JNK(T183/Y185), p38, p‐p38(T180/Y182), p21, p53, Rb, p‐Rb(S780), E2F1, and rabbit IgG were purchased from Cell Signaling Technology (Danvers, MA, USA). Antibodies against importin‐3 and importin‐7 were obtained from Novus (Littleton, CO, USA). HRP‐conjugated secondary antibodies against rabbit IgG and mouse IgG were purchased from EarthOx. Alexa Fluor 488/568 conjugated secondary antibodies against rabbit IgG and mouse IgG were obtained from Thermo Fisher.

### 2.3 Cell culture

HepG2 and 293T cells were obtained from American Type Culture Collection (ATCC, Manassas, VA, USA) and MHCC‐97H cells were purchased from the Liver Cancer Institute (Zhongshan Hospital, Fudan University, China). HL‐7702, PLC/PRF/5, and Huh7 cells were obtained from Shanghai Cancer Institute (Shanghai, China). The cells were cultured in Dulbecco's modified Eagle's medium supplemented with 10% FBS in 5% CO_2_ at 37°C. The cell lines were authenticated by STR profiling at Cobioer Bioscience Co., Ltd. (Nanjing, China), and experiments were performed within <10 passages after authentication.

### 2.4 Clinical HCC sample analysis

For analysis of the correlation between GP73 and MMP‐2 expression, pathological tissues (C, n = 30), and adjacent normal liver tissues (N, n = 30) were freshly obtained from HCC patients undergoing surgery at the First Affiliated Hospital, Zhejiang University School of Medicine (Hangzhou, China). The institutional review board at Zhejiang University School of Medicine approved the protocol of this study, and all patients provided informed consent. The samples were fixed in 4% formalin, routinely processed, and embedded in paraffin. Sections of 4 μm were placed on glass slides for H&E staining and immunohistochemical analysis. Primary antibodies against GP73 were obtained from Thermo Fisher and antibodies against MMP‐2 were purchased from Abcam. A portion of the samples was assessed for differentiation stages in the Department of Pathology, the First Affiliated Hospital, Zhejiang University School of Medicine. For the analysis of serum GP73 and MMP‐2, samples from HCC patients (HCC, n = 40) were derived from the Department of Liver Surgery, the First Affiliated Hospital, Zhejiang University School of Medicine before the surgery, and samples from healthy control individuals (healthy, n = 20) were derived from the Clinical Laboratory in the First Affiliated Hospital, Zhejiang University School of Medicine. The levels of secreted GP73 were measured using the Human GOLM1/GP‐73 ELISA Kit (Raybiotech, Norcross, GA, USA), and the levels of secreted MMP‐2 were measured using the Human MMP‐2 ELISA Kit (Raybiotech) following the manufacturer's instructions.

### 2.5 Small interfering RNA (siRNA) knockdown of human GP73, MMP‐2, and Src

Specific siRNAs for human GP73, MMP‐2, and negative control (siNC) were synthesized by Oligobio (Beijing, China). Specific siRNA pools for Src and siNC were purchased from GE Healthcare Dharmacon Inc.(Lafayette, CO, USA). The sequences of all siRNAs are shown in Table S1A. The cells were transfected with siRNAs using Lipofectamine RNAi Max for 0‐72 hours according to the manufacturer's instructions.

### 2.6 Immunoblotting

For whole‐cell protein extraction, the cells were lysed in RIPA lysis buffer (Merck‐Millipore, Billerica, MA, USA) for 30 minutes on ice and boiled for 5 minutes. For cytosol and nuclear protein extraction, whole cells were lysed using the Nuclear Extract Kit (Active Motif, Carlsbad, CA, USA) following the manufacturer's instructions. The samples were subjected to immunoblotting as described previously.^27^

### 2.7 Immunofluorescence and confocal microscopy

Each well of a 24‐well plate was seeded with 2 × 10^4^ cells and incubated for 24 hours. The remaining procedures were performed as previously reported.^23^

### 2.8 Transwell cell invasion assay

The cell invasion assay was performed using a 6.5 mm Transwell plate with 8.0 μm pore polycarbonate membrane‐coated inserts (Corning, NY, USA). The upper chambers were coated with 15% Matrigel from Corning. The cell invasion assay was performed as described.^28^

### 2.9 Anchorage‐independent cell colony formation assay

Anchorage‐independent proliferation was examined in MHCC‐97H cell lines following GP73 silencing. For this assay, the cells were seeded onto 6‐well plates (1 × 10^4^ cells/mL) and transfected with siGP73s. The cells were digested immediately after transfection and processed as previously described.^29^ The mixture was then incubated at 37°C and 5% CO_2_ for 6 days, and the colonies were counted.

### 2.10 Fluorescence resonance energy transfer

For the fluorescence resonance energy transfer (FRET) assay, the primers sequences for GP73 and MMP‐2 expression are shown in Table S1B. The forward and reverse primers were annealed and cloned into pcDNA3.1‐CFP (GP73) and pcDNA3.1‐YFP (MMP‐2) vectors through HindIII and SacI sites. 293T cells were seeded onto 24‐well plates and transfected with 4 μg of plasmid in proportions of 1:1 using Lipofectamine 3000. Forty‐eight hours later, protein‐protein interactions were examined using a Nikon A1 confocal microscope (Nikon Corporation, Tokyo, Japan) with FRET.

### 2.11 Immunoprecipitation

The cells were lysed in 500 μL of RIPA lysis buffer after reaching approximately 90% confluence in 15 cm dishes. The samples were centrifuged to remove insoluble debris, and the supernatant was split into 2 equal aliquots. Target‐specific antibodies and anti‐rabbit IgG antibodies were added. Immunoprecipitation was performed using Pierce™ Protein A/G Magnetic Beads (Thermo Fisher) according to the manufacturer's instructions.

### 2.12 Isolation of vesicles and exosomes

For vesicle isolation, cells cultured in 15 cm dishes were digested and centrifuged at 1000× *g* for 5 minutes. The pellet was resuspended in 1 mL 1× PBS and ground using a homogenizer. The homogenate was centrifuged at 2000× *g* for 5 minutes. The supernatant was collected and the vesicles were isolated by PEG6000 and ultracentrifugation as previously described.^30^ For exosome isolation, the cells were cultured using serum‐free DMEM for 24 hours in 5% CO_2_ at 37°C. Cell culture media were collected, and the exosomes were isolated using the Exosome Isolation Kit (Thermo Fisher) following the manufacturer's instructions.

### 2.13 Live‐cell imaging

The 293T cells were plated onto glass‐bottom 2.5 cm dishes and transfected with pCMV‐GOLM1‐GFP and pCMV‐MMP2‐OFP (plasmids were purchased from Sinobiological Industries (Beijing, China). Forty‐eight hours after transfection, the movement track of fusion proteins was examined using Nikon A1R confocal microscope (Nikon Corporation). Images were captured every 5 seconds for 10 minutes.

### 2.14 Mapping of the binding site of GP73/MMP‐2 in vitro

Truncated mutants were constructed based on the template of pCMV3‐GOLM1‐flag. PCR was performed with the primers shown in Table S1C. Truncated mutants and pCMV‐MMP2 were transfected into 293T cells. Immunoprecipitation assays were performed as previously described.^23^

### 2.15 Chromatin immunoprecipitation

Chromatin immunoprecipitation (ChIP) analysis was performed using the SimpleChIP Enzymatic Chromatin IP Kit (Cell Signaling Technology) following the manufacturer's instructions. DNA‐protein complexes were precipitated using a specific antibody against E2F1. Immunoprecipitated DNA fragments and input DNA were used as templates for chromatin immunoprecipitation and PCR (ChIP‐PCR) using PrimeSTAR GXL (TaKaRa). The primers used in the ChIP‐PCR analysis are listed in Table S1D.

### 2.16 Luciferase reporter assay

HepG2 cells were seeded onto 24‐well plates and transfected with siNC or siGP73#1. The cells were cotransfected with pGL4.19‐*MMP2*‐promoter, binding site mutated mutants (as shown in Figure 6E) and pRL‐TK for 24 hours before harvest. Luciferase activity was then analysed using the Dual Luciferase Reporter Assay System (Promega, Madison, WI, USA) according to the manufacturer's instructions. Total light production was measured with the SpectraMax M5 multimode microplate reader (Molecular Devices, Sunnyvale, CA, USA).

### 2.17 Statistical analysis

The values were analysed by two‐tailed Student's *t*‐test and demonstrated as the means ± standard error of the mean (SEM). Statistical analysis was performed using Statistical Package for Social Sciences (SPSS) software (version 16.0, IBM Corporation, Armonk, NY, USA).

## 3 RESULTS

### 3.1 GP73 correlates positively with MMP‐2 in tissues and serum derived from HCC patients

**Figure 1:**
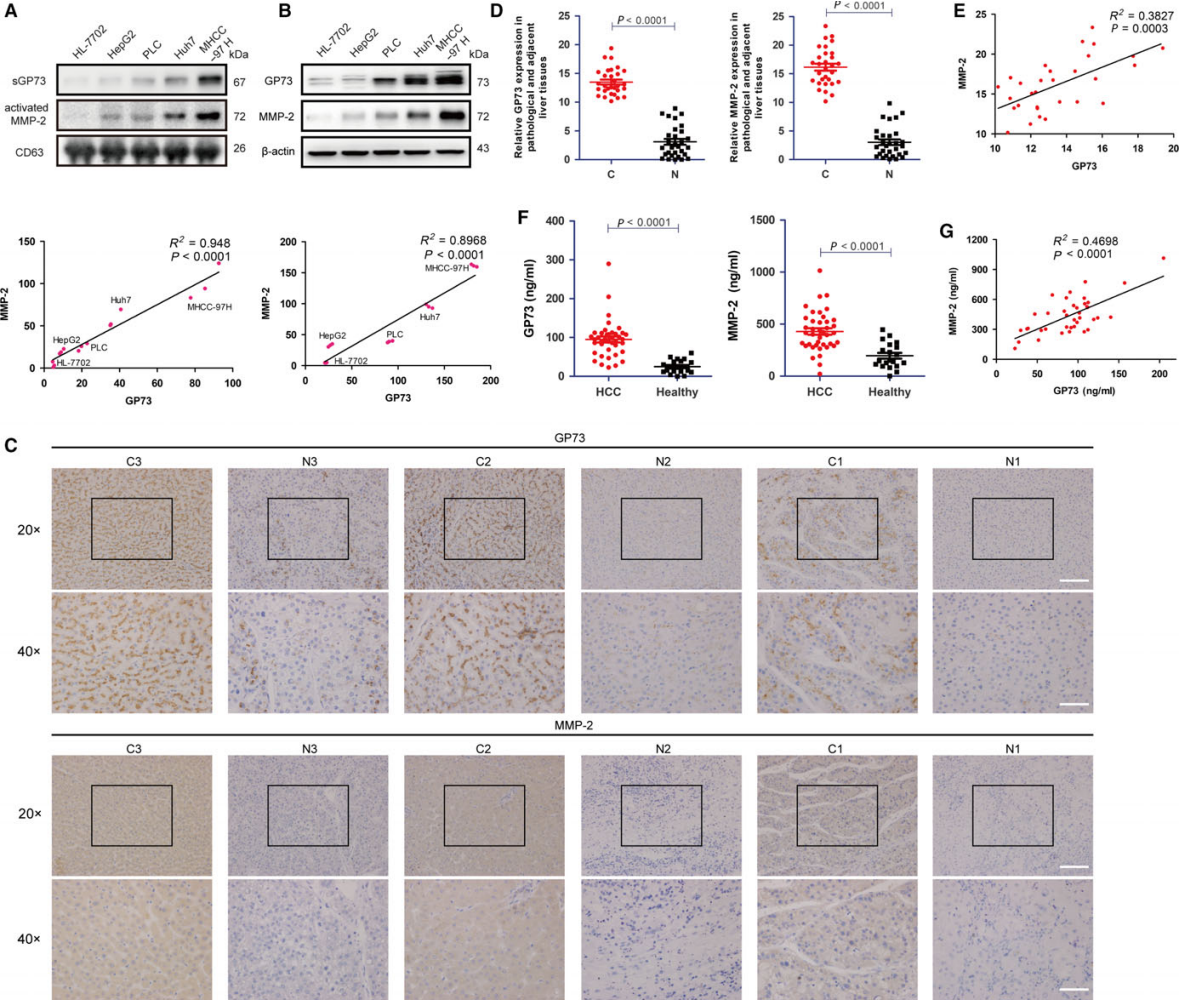
GP73 correlates positively with MMP‐2 in tissues and serum derived from HCC patients. (A) Immunoblot analysis of sGP73 and activated MMP‐2 in the exosomes of five normal and liver cancer cell lines. (B) Immunoblot analysis of intracellular GP73 and MMP‐2 in the cell lysates of five normal and liver cancer cell lines. (C) Immunohistochemical analysis of GP73 and MMP‐2 in pathological (C, n = 30) and adjacent liver (N, n = 30) tissues from HCC patients. Scale bar, 60 μm (20×) and 30 μm (40×). (D) Data in c were evaluated using average optical density (AOD). AOD values in the pathological tissues group were compared with those in the adjacent liver tissues group. (E) Abundance and correlation of GP73 and MMP‐2 in pathological tissues from HCC patients were analysed. (F) ELISA of GP73 and MMP‐2 in serum derived from HCC patients (HCC, n = 40) and individuals under physical examination (healthy, n = 20). GP73 and MMP‐2 values in the HCC patient group were compared with those in the physical examination group. (G). Abundance and correlation of GP73 and MMP‐2 in the serum of HCC patients were analysed. The data in A, B, and D‐G are presented as the means ± SEM, and the data in A and B are representative of three independent experiments. Two‐tailed Student's *t*‐test was used for statistical calculation

To determine how GP73 facilitates cell invasion, we analysed the expression of secretory proteins in the exosomes of five different normal or liver cancer cell lines (R^2^ = 0.948) and confirmed that secretory GP73 (sGP73) correlated positively with cytosolic MMP‐2 (Figure 1A). Expression levels of cytosolic GP73 and MMP‐2 were also measured using immunoblot analysis, and these proteins demonstrated a correlation with each other (R^2^ = 0.8968) (Figure 1B), which suggested that the transcription of *GOLM1* and *MMP2* might be associated.

To confirm the clinical correlation of these two factors, intracellular GP73 and MMP‐2 were detected by immunohistochemical analysis in pathological and adjacent liver tissues obtained from 30 HCC patients, and the results were evaluated by average optical density (AOD) (Figure 1C). The results indicated that both GP73 and MMP‐2 were highly expressed in pathological tissues compared with adjacent liver tissues (Figure 1D). Linear regression analysis showed that GP73 correlated positively with MMP‐2 (R^2^ = 0.3827) (Figure 1E), which indicated that GP73 was highly correlated with MMP‐2 in HCC tissues. Because secretory GP73 and MMP‐2 facilitated cell invasion through secretion into the extracellular matrix and serum, it was essential to measure serum GP73 and MMP‐2. The content of extracellular GP73 and MMP‐2 in serum derived from 40 HCC patients and 20 individuals under physical examination were detected using ELISA. Similarly, GP73 and MMP‐2 in the serum of HCC patients were comprehensively higher than in the serum of individuals under physical examination (Figure 1F). Strikingly, the correlation of GP73 and MMP‐2 was closer in serum than that in HCC tissues (R^2^ = 0.4698) (Figure 1G). The data above imply that the expression of GP73 might be associated with the generation or secretion of MMP‐2.

### 3.2 Knockdown of GP73 blocks trafficking of intracellular MMP‐2 and inhibits cell invasion

**Figure 2:**
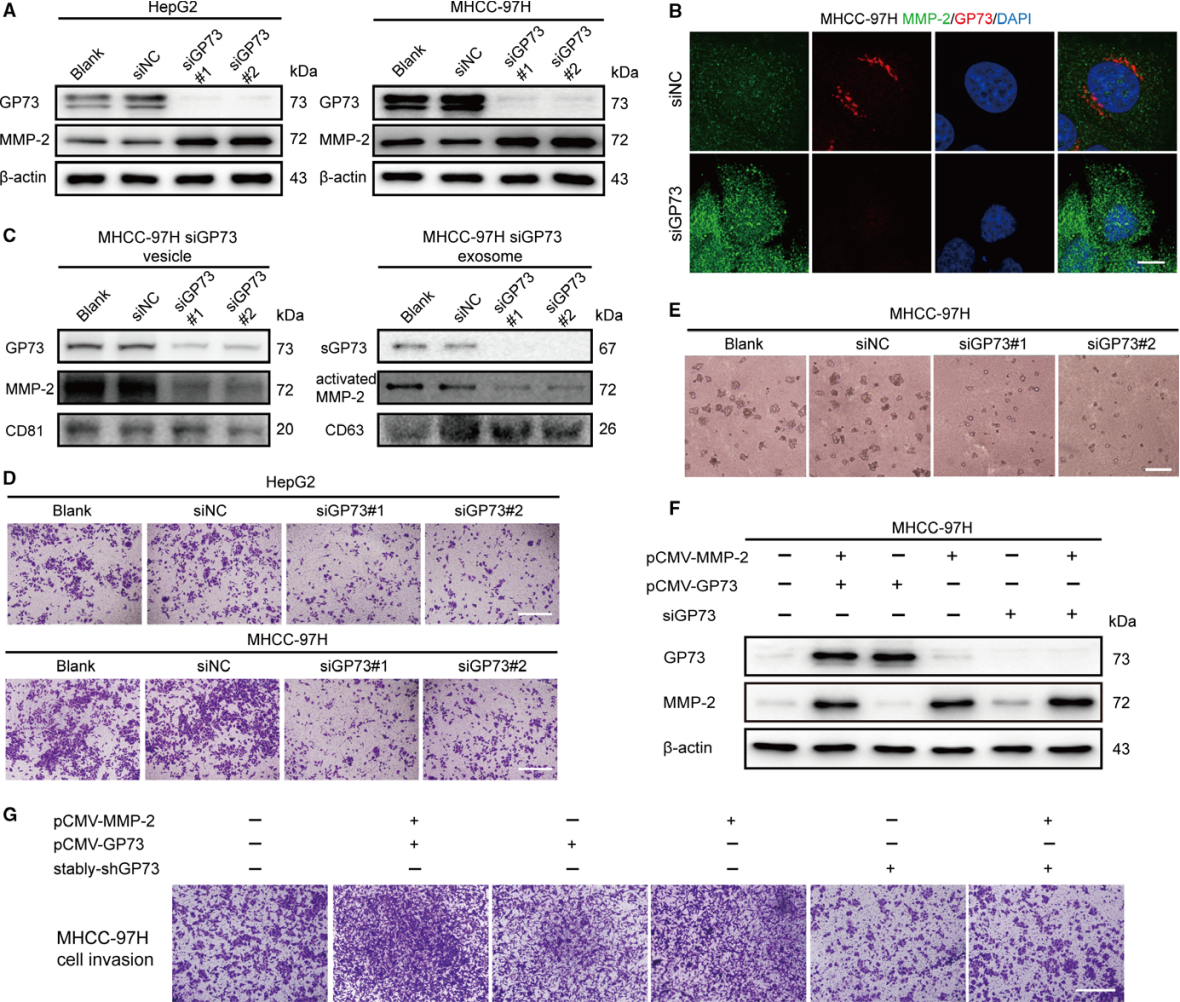
Knockdown of GP73 inhibits cell invasion. (A) Immunoblot analysis of intracellular GP73 and MMP‐2 in HepG2 and MHCC‐97H cells transfected for 24 h with siGP73s (50 nmol L^−1^). (B) Immunofluorescence and confocal microscopy analysis of the colocalization of intracellular MMP‐2 (green) and GP73 (red) in HepG2 cells transfected for 24 h with siGP73s. Scale bar, 5 μm. (C) Immunoblot analysis of sGP73 and MMP‐2 in the exosomes of MHCC‐97H cells transfected for 24 h with siGP73s. (D) Matrigel‐Transwell invasion assay of HepG2 and MHCC‐97H cells transfected for 24 h with siGP73s. Scale bar, 100 μm. (E) Colonies of MHCC‐97H cells were captured at 6 d after transfection of siGP73s. Scale bar, 200 μm. (F) Immunoblot analysis of endogenous and exogenous GP73 and MMP‐2 in MHCC‐97H cells transfected for 48 h with pCMV3‐GOLM1‐flag or pCMV‐MMP‐2. The cells were pretreated with siGP73 or siNC for 24 h before plasmid transfection. The proteins were detected using anti‐GP73 and anti‐MMP‐2 to recognize total GP73 and MMP‐2. (G) Transwell invasion assay of MHCC‐97H cells transfected with siGP73s and the plasmids described in (F) The cells were collected and seeded onto Transwell chambers at 6 h after plasmid transfection

To investigate the association of GP73 and MMP‐2, we silenced endogenous GP73 in HepG2 and MHCC‐97H cells by using two pairs of GP73‐specific siRNAs (siGP73s). Immunoblotting and immunofluorescence assays showed that the knockdown of GP73 upregulated intracellular MMP‐2 (Figure 2A, B). However, the expression of vesicular and exosomal MMP‐2 was reduced (Figure 2C). Transwell invasion and anchorage‐independent cell colony formation assays demonstrated that cell invasion was silenced after GP73 silencing (Figure 2D, E), which suggested that the knockdown of GP73 might inhibit the trafficking and secretion of MMP‐2. To further determine whether GP73 silencing blocked MMP‐2 trafficking, MMP‐2 was overexpressed in MHCC‐97H^siGP73^ cells, but strikingly, cell invasion was still inhibited, which further indicated that GP73 is involved in the trafficking of intracellular MMP‐2 (Figure 2F, G).

**Figure 3:**
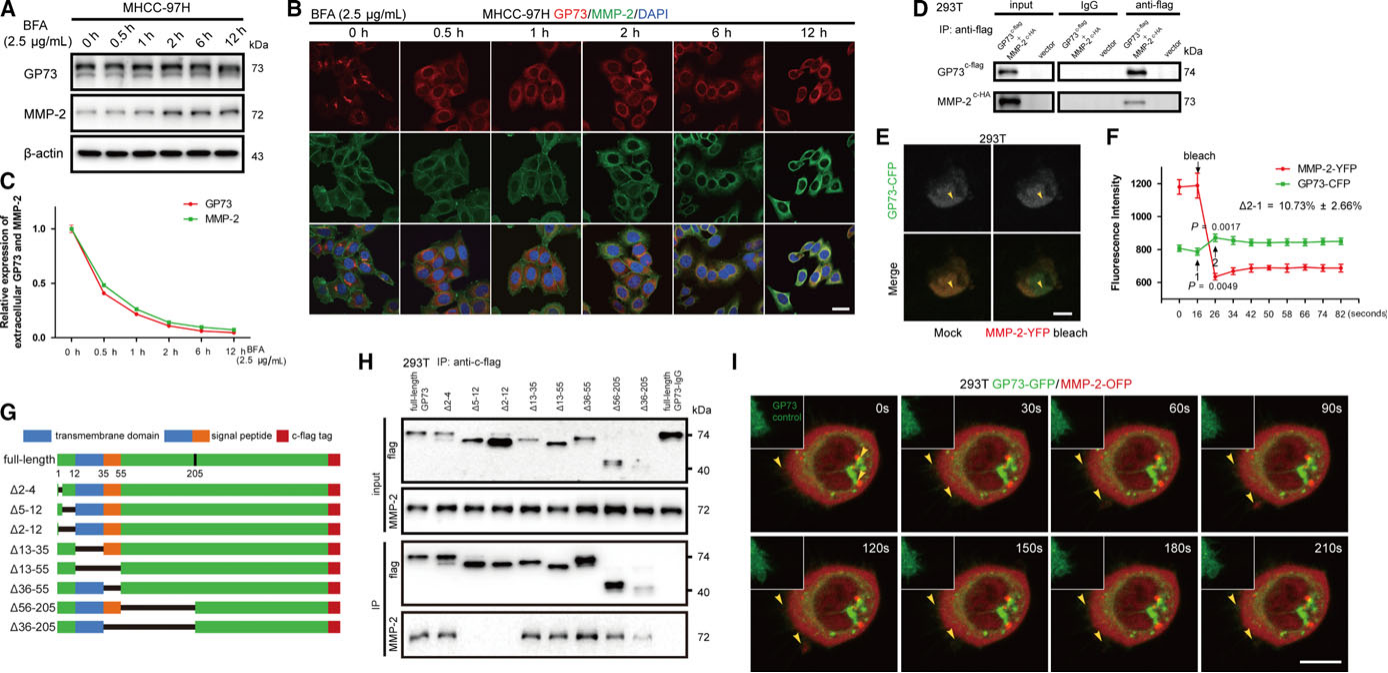
GP73 is involved in MMP‐2 trafficking. (A) MHCC‐97H cells were treated with BFA (2.5 μg/mL) for 0, 0.5, 1, 2, 6, and 12 h. The expression of GP73 and intracellular MMP‐2 was measured using immunoblotting. (B) GP73 (red) and intracellular MMP‐2 (green) in MHCC‐97H cells were detected using immunofluorescence and confocal microscopy after treatment with BFA. Scale bar, 10 μm. (C) MHCC‐97H cells were treated with BFA (2.5 μg/ml) for 0, 0.5, 1, 2, 6, and 12 h, and cell culture media were collected. The expression of extracellular GP73 and MMP‐2 was determined using ELISA. (D) Coimmunoprecipitation and immunoblot analysis of GP73‐c‐flag and MMP‐2‐c‐HA in 293T cells transfected for 48 h with pCMV3‐GOLM1‐flag and pCMV‐MMP‐2‐HA. The proteins were immunoprecipitated using Protein A/G beads conjugated with anti‐flag antibody. (E) Fluorescence resonance energy transfer (FRET) analysis of the interaction of GP73‐CFP and MMP‐2‐YFP fusion proteins. The fluorescence of YFP was bleached, and the energy shift of CFP was measured. Scale bar, 5 μm. (F) Fluorescence intensity of CFP increased 10.73±2.66%, which demonstrated that GP73 interacted directly with intracellular MMP‐2. (G) Diagrammatic representation of *GOLM1* and the truncated forms. (H) Coimmunoprecipitation and immunoblot analysis of GP73 deletion mutants and intracellular MMP‐2 in 293T cells transfected for 48 h with pCMV3‐GOLM1‐flag deletion mutants. The proteins were immunoprecipitated using Protein A/G beads conjugated with anti‐flag antibody. (I) Live cell imaging of GP73‐GFP (green) and MMP‐2‐OFP (red) in 293T cells. The arrowheads indicate the colocalization of GP73 and MMP‐2 in the Golgi apparatus and extracellular vesicles. Scale bar, 10 μm. The data in F are presented as the means ± SEM and are representative of three independent experiments. Two‐tailed Student's *t*‐test was used for statistical calculation

To further confirm the trafficking relationship between GP73 and MMP‐2, MHCC‐97H cells were cultured in DMEM containing Brefeldin A (BFA, 2.5 μg/mL). Immunoblotting and immunofluorescence analysis demonstrated that GP73 was secreted into the cytosol and that MMP‐2 accumulated in the cytosol, but extracellular MMP‐2 was reduced, which implied that GP73 might play important roles in MMP‐2 trafficking while the Golgi apparatus was intact. Twelve hours after BFA stimulation, GP73 overlapped with MMP‐2 in the cytosol, which suggested that GP73 potentially interacted with MMP‐2 (Figure 3A‐C). Coimmunoprecipitation assays proved that exogenous GP73 interacted with MMP‐2, and fluorescence resonance energy transfer (FRET) assays further determined that GP73 directly interacted with MMP‐2 in the cytosol (Figure 3D‐F). The evidence above demonstrated that GP73 directly participated in the trafficking of intracellular MMP‐2. To determine the binding site of GP73 and intracellular MMP‐2, a series of constructs encoding *GOLM1* deletion mutants with c‐flag tags were constructed (Figure 3G). The deletion constructs and pCMV‐MMP‐2 were cotransfected into 293T cells, followed by coimmunoprecipitation and immunoblot analysis. Almost all of the GP73 deletion mutants interacted with exogenous MMP‐2, except for the Δ5‐12 and Δ2‐12 mutants, which proved that GP73 interacted with intracellular MMP‐2 in the region of the cytoplasmic domain (Figure 3H). These results implied that GP73 interacted with MMP‐2 and participated in MMP‐2 trafficking by vesicular transport. To track the process of MMP‐2 trafficking, GP73‐GFP and MMP‐2‐OFP fusion proteins were expressed in 293T cells, and live cell imaging displayed that GP73 and MMP‐2 partially overlapped in the region of the Golgi apparatus, both factors translocated to the plasma membrane and were secreted into extracellular spaces (Figure 3I).

The data above show that GP73 interacts with intracellular MMP‐2 in the region of the cytoplasmic domain, facilitating the trafficking and secretion of MMP‐2. Knockdown of GP73 blocks MMP‐2 trafficking and inhibits cell invasion.

### 3.3 Knockdown of GP73 inhibits SAPK/JNK and p53‐p21 signalling pathways by a negative feedback loop

**Figure 4:**
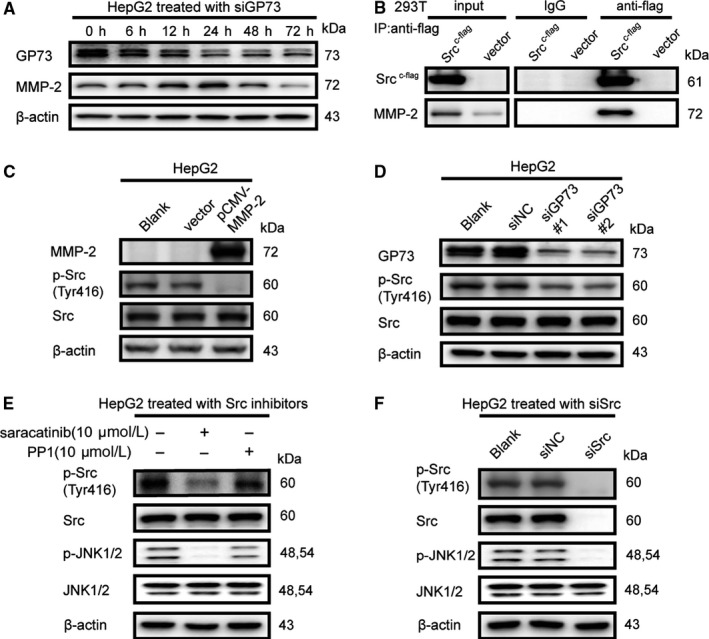
Accumulation of intracellular MMP‐2 inhibits phosphorylation of Src at Y416. (A) Immunoblot analysis of GP73 and intracellular MMP‐2 in HepG2 cells transfected for 0, 6, 12, 24, 48, and 72 h with siGP73#1. (B) Coimmunoprecipitation and immunoblot analysis of Src‐c‐flag and endogenous MMP‐2 in 293T cells transfected for 48 h with pCMV‐Src‐c‐flag. The proteins were immunoprecipitated using Protein A/G beads conjugated with anti‐flag antibody. (C) Immunoblot analysis of MMP‐2 and p‐Src (Y416) in HepG2 cells transfected for 48 h with p‐CMV‐MMP‐2. (D) Immunoblot analysis of GP73, MMP‐2, and p‐Src (Y416) in HepG2^siGP73^ cells compared with siNC cells. (E) Immunoblot analysis of p‐JNK1/2 (T183/Y185) in HepG2 cells treated with saracatinib (10 μmol L^−1^, 12 h) and PP1 (10 μmol L^−1^, 2 h). (F) Immunoblot analysis of p‐JNK1/2 (T183/Y185) in HepG2 cells transfected for 48 h with siSrc (siRNA pool, 50 nmol L^−1^)

The knockdown of GP73 induces the accumulation of intracellular MMP‐2. To further investigate this effect, MHCC‐97H cells were transfected with siGP73s, and cell lysates were collected at 0‐72 hours after transfection. Immunoblotting analysis showed that intracellular MMP‐2 increased at 24 hours after transfection because MMP‐2 trafficking was inhibited. However, the expression of intracellular MMP‐2 was reduced at 48 and 72 hours after transfection, which suggested that the transcription of *MMP2* might be inhibited to maintain the equilibrium of intracellular MMP‐2 (Figure 4A).

**Figure 5:**
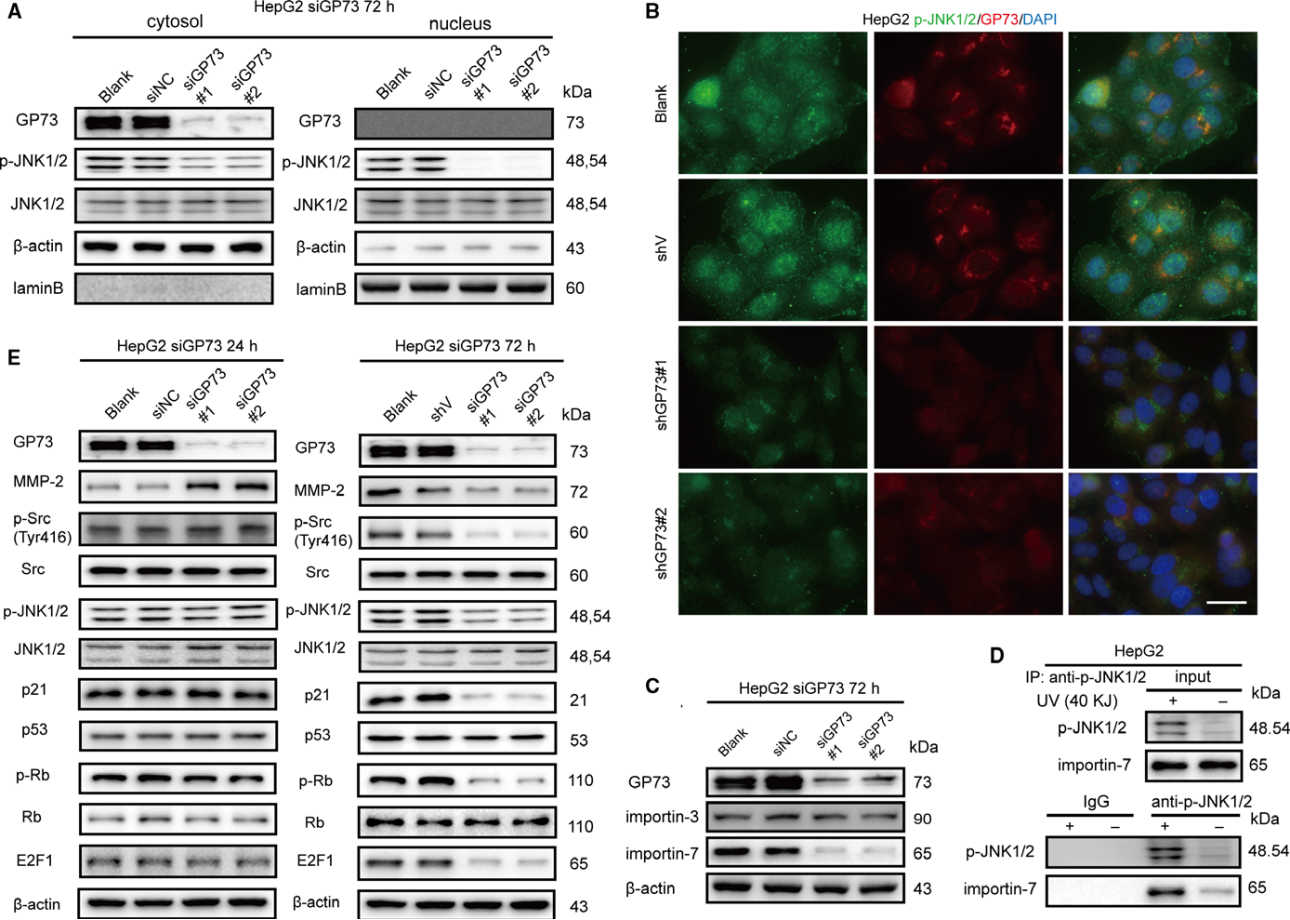
Knockdown of GP73 inhibits the SAPK/JNK and p53‐p21 signalling pathways by a negative feedback loop. (A) Immunoblot analysis of p‐JNK1/2 (T183/Y185) in the nucleus and cytosol of HepG2^siGP73^ cells compared with that in siNC cells. (B) Immunofluorescence and confocal microscopy analysis of the colocalization of p‐JNK1/2 (T183/Y185) (green) and GP73 (red) in HepG2^siGP73^ cells compared with that in siNC cells. Scale bar, 10 μm. (C) Immunoblot analysis of importin‐3 and importin‐7 in HepG2^siGP73^ cells compared with that in siNC cells. (D) Coimmunoprecipitation and immunoblot analysis of p‐JNK1/2 (T183/Y185) and importin‐7 in 293T cells treated with UV (40KJ). The proteins were immunoprecipitated using Protein A/G beads conjugated with anti‐p‐JNK1/2 (T183/Y185) antibody. (E) Immunoblot analysis of key factors in the SPAK/JNK and p53‐p21 signalling pathways in HepG2^siGP73‐72 h^ cells compared with those in HepG2^siGP73‐24 h^ cells

Intracellular MMP‐2 was purified using immunoprecipitation, and MMP‐2‐interacting proteins were identified using LC‐MS/MS. Src interacted with intracellular MMP‐2. As previously reported,^31^ p‐Src (Y416) modulated the expression of MMP‐2 to promote cell invasion, and herein, we hypothesized that accumulated intracellular might inhibit the phosphorylation of Src at Y416. Src‐c‐flag fusion protein was expressed in 293T cells, and whether MMP‐2 interacted with Src was determined using coimmunoprecipitation and immunoblotting analysis (Figure 4B). To further confirm our previous hypothesis, exogeneous MMP‐2 was overexpressed in HepG2 cells, revealing that the phosphorylation of p‐Src (Y416) was inhibited (Figure 4C). Further evidence demonstrated that the phosphorylation of p‐Src (Y416) was inhibited at 72 hours after GP73 silencing, which proved that the accumulation of intracellular MMP‐2 indeed inactivated the phosphorylation of p‐Src (Y416) (Figure 4D). To inactivate the phosphorylation of p‐Src (Y416), HepG2 cells were treated with a Src‐specific inhibitor or siRNAs, and the results showed that the phosphorylation of p‐JNK1/2 (T183/Y185) was inhibited (Figure 4E, F). Because p‐JNK1/2 (T183/Y185) functions as a protein kinase by translocating to the nucleus,^32^ we measured the expression of cytosolic and nuclear p‐JNK1/2 (T183/Y185) after HepG2 cells were transfected with siGP73 for 72 hours. Surprisingly, the knockdown of GP73 not only inhibited the phosphorylation of p‐JNK1/2 (T183/Y185) but also blocked its nuclear translocation (Figure 5A, B). Importin‐3 and importin‐7 participated in the nuclear translocation of p‐JNK1/2 (T183/Y185),^33^ and it was found that expression of importin‐7 was reduced in HepG2^siGP73^ groups compared with siNC group (Figure 5C). Coimmunoprecipitation and immunoblotting analysis further verified that p‐JNK1/2 (T183/Y185) interacted with importin‐7 (Figure 5D).^34^

Because the nuclear translocation of p‐JNK1/2 (T183/Y185) was inhibited, p53‐p21 signalling pathways were blocked, and the phosphorylation of p‐Rb (S780) was inactivated, which led to a reduction in the expression of the transcription factor E2F1 (Figure 5E).^35^, ^36^

### 3.4 Reduction in E2F1 inhibits transactivation of *MMP2*

**Figure 6:**
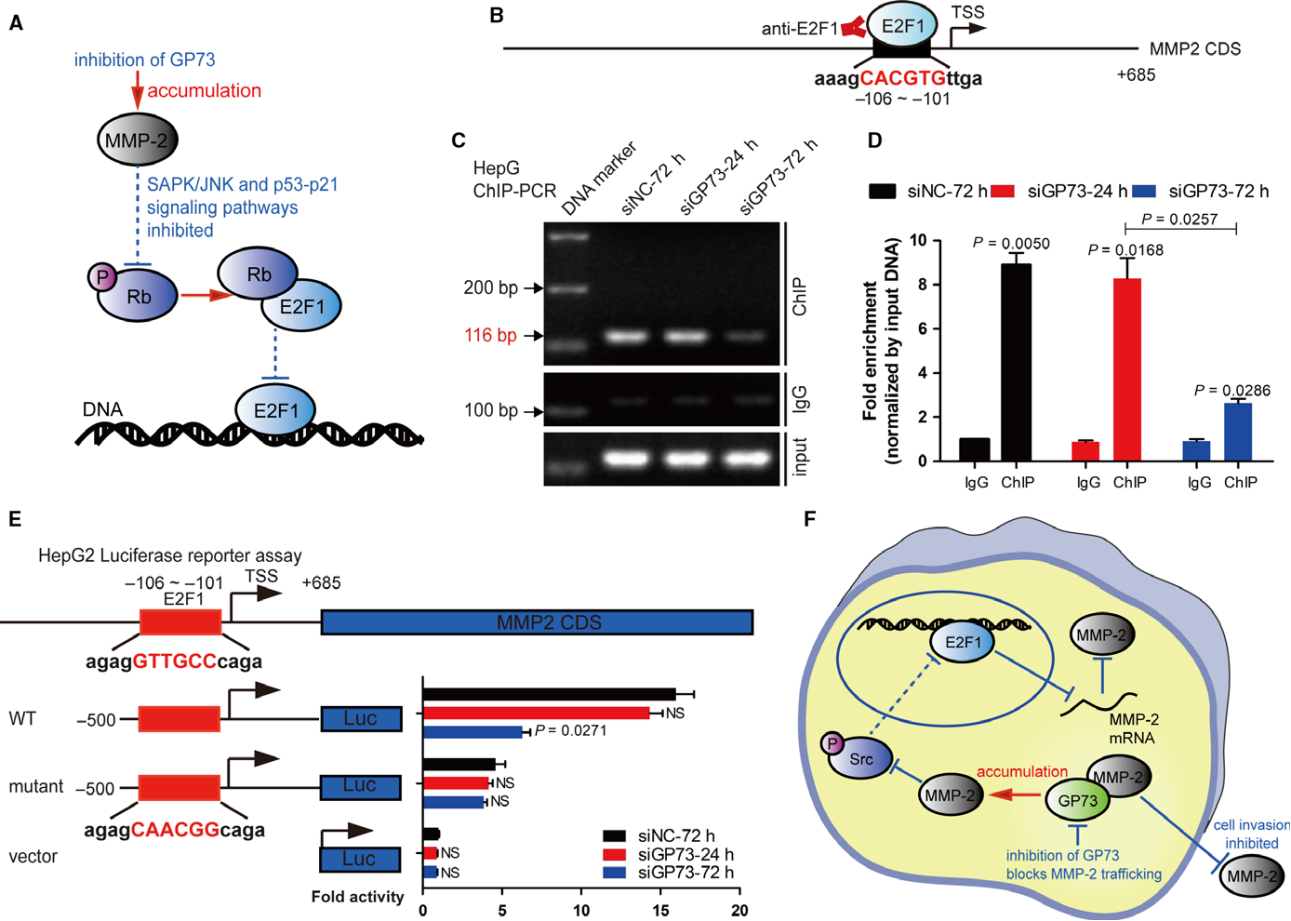
Reduction in E2F1 inhibits the transactivation of *MMP2*. (A) Schematic representation of the modulative mechanisms of the Rb‐E2F1 complex after GP73 silencing for 72 h. (B) Schematic representation of the promoter and 5′UTR region of human *MMP2*. The predicted E2F1 binding site is indicated. (C) ChIP‐PCR analysis of the predicted binding site on the *MMP2* promoter in HepG2^siGP73‐72 h^ cells compared with that in HepG2^siGP73‐72 h^ cells. The amplification products were analysed using agarose electrophoresis analysis. (D) Fold enrichment of amplification products derived from ChIP‐PCR analysis. (E) Luciferase activities of HepG2 cells cotransfected with siGP73, pGL4.19‐*MMP2*‐promoter, binding site mutated mutants and pRL‐TK for 24 or 72 h. (F) Overview of the negative feedback loop. The knockdown of GP73 for 72 h inhibited the SAPK/JNK and p53‐p21 signalling pathways, resulting in reduced of *MMP2* transcriptional activity and inhibited cell invasion. The data in D and E are presented as the means ± SEM and are representative of three independent experiments. Two‐tailed Student's *t*‐test was used for statistical calculation

As mentioned above, the accumulation of intracellular MMP‐2 activated a negative feedback loop and blocked the expression of factors in the SAPK/JNK and p53‐p21 signalling pathways, leading to the inhibition of p‐Rb phosphorylation (S780). As previously reported, the inactivation of p‐Rb (S780) promoted the formation of the Rb‐E2F1 complex and reduced the content of free E2F1, indicating that the transactivation effect of E2F1 might be inhibited after GP73 silencing (Figure 6A).^37^, ^38^ Moreover, E2F1, 2, and 3 serve as transcription factors for *MMP*9, *MMP14,* and *MMP15,* 39 which suggested that E2F1, as a member of the E2F family, might regulate the transcription of *MMP2* in similar ways. A potential E2F1 binding site located in the promoter of *MMP2* was found, and a chromatin immunoprecipitation (ChIP) assay was performed in HepG2 cells to verify this hypothesis (Figure 6B). Chromatin immunoprecipitation and PCR (ChIP‐PCR) analysis showed that E2F1 interacted with the promoter of *MMP2* at the ‐106/‐101 site. The fold enrichment decreased in the siGP73‐72 hours group compared with that in the siGP73‐24 group, which proved that the negative feedback loop was activated and inhibited the transcription of *MMP2* (Figure 6C, D). Additionally, luciferase reporter analysis further demonstrated that transcriptional activity was inhibited in the siGP73‐72 hours group, which is consistent with the results derived from the ChIP analysis (Figure 6E).

The data above proved that accumulated intracellular MMP‐2 activated a negative feedback loop and inhibited the SPAK/JNK and p53‐p21 signalling pathways after GP73 silencing, resulting in the inhibition of *MMP2* transactivation (Figure 6F). In this way, the equilibrium of intracellular MMP‐2 was maintained.

## 4 DISCUSSION

In recent years, 90% of HCC‐related mortality incidences correlate with metastasis. The mechanism of HCC metastasis remains complex. GP73, as a serum biomarker, has been comprehensively used in the diagnosis of HCC in recent years and has been associated with the carcinogenesis and metastasis of HCC. The knockdown of GP73 inhibited the expression of EMT‐related factors, such as MMP‐7, CD44 and EGFR.^8^, ^23^ However, the regulating and coordinating mechanisms are still poorly understood.

Because GP73 is located in the *cis*‐Golgi apparatus, mechanistically, it is supposed that highly expressed GP73 might promote the metastasis of HCC through regulating the trafficking of EMT‐related membrane and secretory proteins. Therefore, in our preliminary work, the content of GP73 and other EMT‐related proteins was measured in exosomes derived from 5 normal or liver cancer cell lines to discover a dominant EMT‐related secretory protein closely correlated with GP73. Fortunately, GP73 correlated positively with MMP‐2 in the exosomes (R^2^ = 0.948) derived from these cell lines, which implied that GP73 might participate in MMP‐2 trafficking and secretion. In follow‐up studies, the knockdown of GP73 induces the accumulation of intracellular MMP‐2 but inhibits MMP‐2 secretion. These results confirm that GP73 plays important roles in MMP‐2 trafficking. Subsequent results indicated that GP73 interacts directly with intracellular MMP‐2 in the region of the cytoplasmic domain and assists in the trafficking and secretion of MMP‐2.

However, the knockdown of GP73 blocks MMP‐2 trafficking and induces the accumulation of intracellular MMP‐2. The accumulation of MMP‐2 inhibits the phosphorylation of p‐Src (Y146) and blocks the SPAK/JNK signalling pathway. Moreover, importin‐7 was reduced in HepG2^siGP73^ cells using the iTRAQ assay, which blocks the nuclear translocation of p‐JNK1/2 (T183/Y185) and inactivates the factors in p53‐p21 signalling pathways. However, it is difficult to find the reason for the reduction in importin‐7. We deem that the knockdown of GP73 might block the trafficking of other membrane receptors, which inhibits the transcription of *IPO7*. Finally, the transactivation of *MMP2* is suppressed because of the inhibition of E2F1, which maintains the equilibrium of intracellular MMP‐2.

The evidence above reveals that GP73 plays a role in the trafficking and equilibrium of EMT‐related secretory proteins. In addition, we propose that MMP‐2 is not the only trafficking substrate of GP73 because the knockdown of GP73 strongly inhibits cell proliferation and invasion of liver cancer cell lines, which implies that not only the trafficking of MMP‐2 is inhibited. We have identified more trafficking substrates of GP73, and these proteins might be reported in forthcoming studies.

MMPs, especially MMP‐2 and MMP‐9, are highly expressed in pathological tissues derived from patients with metastatic HCC after SIRT treatment.^40^ In recent years, patients with metastatic HCC have been treated with personalized management and tailored therapy.^41^, ^42^, ^43^ GP73 might be a potential target in tailored therapy targeting metastatic HCC.

This study also indicated that GP73 correlates positively with MMP‐2 in serum derived from HCC patients. GP73 is correlated with the process of HCC, and MMP‐2 is correlated with cell invasion. Because these proteins are closely related to each other, it is supposed that they can serve as serum indicators of metastatic HCC. Unfortunately, insufficient serum samples from HCC patients were collected in this study, and it is illogical to prove that these proteins can serve as proper biomarkers in the diagnosis of metastatic HCC. However, we propose that serum GP73 and MMP‐2 can be excellent serum indicators for the diagnosis of metastatic HCC.

In summary, this study indicates that GP73 plays critical roles in MMP‐2 trafficking. The knockdown of GP73 blocks the secretion of MMP‐2 and suppresses the transactivation of *MMP2* by inhibiting the SAPK/JNK and p53‐p21 signalling pathways and finally inhibiting cell invasion. This study provides a potential target in HCC therapeutics.

## CONFLICT OF INTEREST

The authors declare that they have no conflict of interest.
