# Supplementary material for: Knockdown of Golgi phosphoprotein 73 blocks the trafficking of matrix metalloproteinase‐2 in hepatocellular carcinoma cells and inhibits cell invasion
Source: J Cell Mol Med. 2019 Jan 24;23(4):2399–409. doi: 10.1111/jcmm.14055 (PMC6433683; doi:10.1111/jcmm.14055)
Supplement: Supplementary file 1 [file JCMM-23-2399-s001.doc]

**Supplementary Figures**

**Figure S1**

**
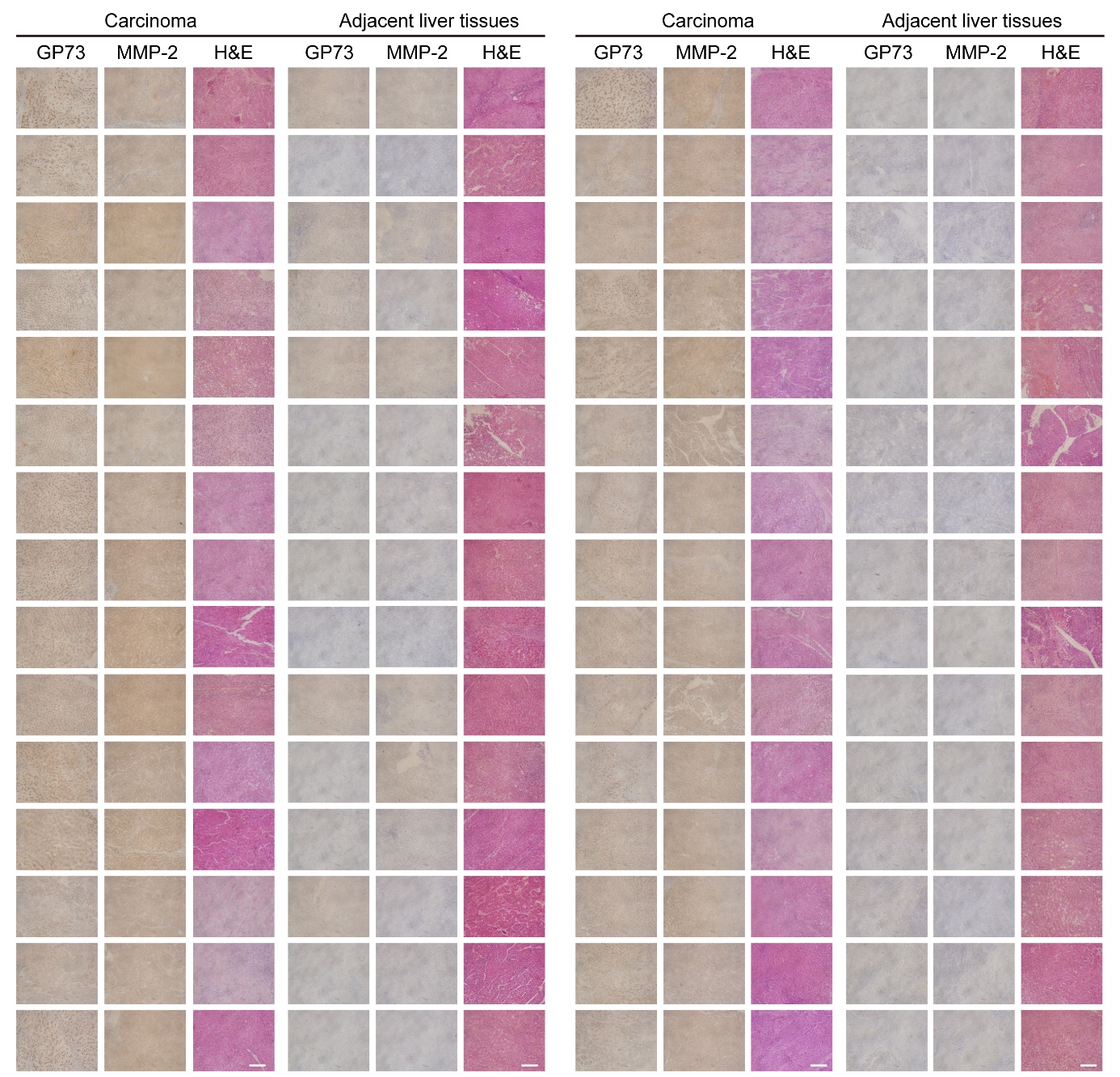
**

**Figure S2**

**
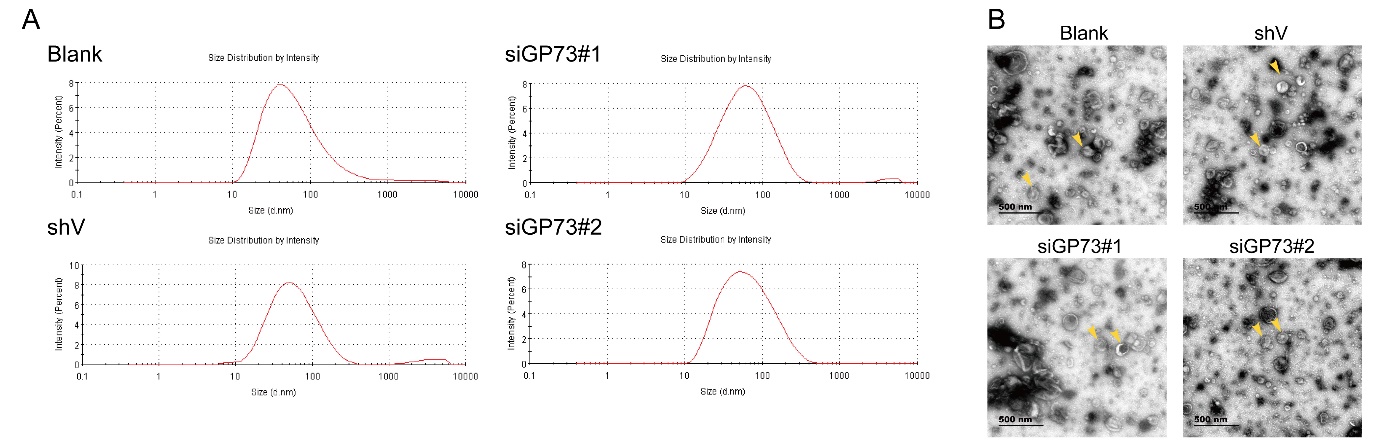
**

**Supplementary legends to figures**

**Figure S1.** GP73 correlates positively with MMP-2 in HCC and adjacent liver tissues. Original data of H&E staining and immunohistochemical assay of the HCC and adjacent liver tissues derived from HCC patients.

**Figure S2. Identification of MHCC-97H derived exosomes.** **A.** Size distribution of exosomes derived from MHCC-97H cells are analyzed through exosome nanoparticle tracking analysis. **B.** Transmission electron microscopy analysis of exosomes secreted from MHCC-97H cells transfected for 48h with siGP73s. Scale bar, 500 nm.

**Supplementary Tables**

**Supplementary Table 1**

A. siRNAs for human GP73, MMP-2 and Src

| Targets Sense and antisense chains (5’→3’) |
| --- |
| GP73#1 S: GUGGCUUAGAAUUUGAACATT  A: UGUUCAAAUUCUAAGCCACTT  GP73#2 S: CAAGCUGUACCAGGACGAATT  A: UUCGUCCUGGUACAGCUUGTT  Src Pool: GCAGUUGUAUGCUGUGGUU  GCAGAGAACCCGAGAGGGA  CCAAGGGCCUCAACGUGAA  GGGAGAACCUCUAGGCACA  NC S: GCGACGAUCUGCCUAAGAUTT  A: AUCUUAGGCAGAUCGUCGCTT |

**­**

B. Primers for FRET assays

| cDNAs Forward and reverse primers (5’→3’) |
| --- |
| GP73 F: CCCAAGCTTATGATGGGCTTGGGAAACG  R: CCGCTCGAGGAGTGTATGATTCCGCTTTTCAC  MMP-2 F: CCCAAGCTTATGGAGGCGCTAATGGCCC  R: CCGCTCGAGGCAGCCTAGCCAGTCGGAT |

C. Primers for mapping of the binding site of GP73/MMP-2 *in vivo*

| Truncated mutant Forward and reverse primers (5’→3’) |
| --- |
| GP73（Δ2-4） F:CGGGGTACCATGGGAAACGGGCGTCGCAGCATG  GP73（Δ2-4） R:CTAGTCTAGACTCGAGTTTACTTATCGTC  GP73（Δ2-12） F:CGGGGTACCATGTCGCCGCCCCTCGTGCTGG  GP73（Δ2-12） R:CTAGTCTAGACTCGAGTTTACTTATCGTC  GP73（Δ5-12） F:TCGCCGCCCCTCGTGCTGG  GP73（Δ5-12） R:CAAGCCCATCAT GGTACC  GP73（Δ13-35） F:AGCTCCCGGAGCGTGGACCT  GP73（Δ13-35） R:CTTCATGCTGCGACGCCCGT  GP73（Δ13-55） F:GCGGCTGCAGAGAGAGGCG  GP73（Δ13-55） R:CTTCATGCTGCGACGCCCGT  GP73（Δ36-55） F:GCGGCTGCAGAGAGAGGCG  GP73（Δ36-55） R:CGCAATCCAGTAGTTGAAGCC  GP73（Δ36-205） F:CCTCAGCCCAGGCTGCAGG  GP73（Δ36-205） R:CGCAATCCAGTAGTTGAAGCC  GP73（Δ56-205） F:CCTCAGCCCAGGCTGCAGG  GP73（Δ56-205） R:CCTGCGGACCCTGCCTTCC |

D. Primers for ChIP assay

| Genes Forward and reverse primers (5’→3’) |
| --- |
| *MMP2*(-106/-101) F: CACCTGCTGTGGGGATGTAG (-162)  R: AAGAAGTGCTGCTTTGCTGC (-47) |
